# Altered Episodic Memory in Introverted Young Adults Carrying the BDNF_Met_ Allele

**DOI:** 10.3390/ijms17111886

**Published:** 2016-11-12

**Authors:** Andreanne Bombardier, Maude Beauchemin, Nadia Gosselin, Judes Poirier, Louis De Beaumont

**Affiliations:** 1 Department of Psychology, University of Quebec at Trois-Rivieres, 3351 Boulevard des Forges, Trois-Rivières, QC G9A 5H7, Canada; andreanne.bombardier@uqtr.ca; 2 Department of Psychology, University of Montreal, 2900 Boulevard Edouard-Montpetit, Montréal, QC H3T 1J4, Canada; maude.beauchemin@umontreal.ca (M.B.); nadia.gosselin@umontreal.ca (N.G.); 3 Montreal Sacred Heart Hospital Research Centre, Montreal, QC H4J 1C5, Canada; 4 Douglas Mental Health University Institute, 6875 Boulevard LaSalle, Verdun, QC H4H 1R3, Canada; judes.poirier@mcgill.ca

**Keywords:** brain-derived neurotrophic factor, personality trait, memory

## Abstract

While most studies have been interested in the distinct, predisposing roles of the common BDNF Val66Met variant and extraversion personality traits on episodic memory, very few studies have looked at the synergistic effects of genetic and personality factors to account for cognitive variance. This is surprising considering recent reports challenging the long-held belief that the BDNF_Met_ variant negatively impacts cognitive function. A total of 75 young healthy adults (26 of them carried at least one copy of the BDNF_Met_ allele) took part in this study consisting of genetic profiling from saliva, personality assessment using the Revised NEO Personality Inventory (NEO PI-R) and a short battery of neuropsychological tests. An ANOVA revealed that BDNF_Met_ carriers were significantly less extraverted than BDNF_Val_ carriers (*F*_1,73_ = 9.54; *p* < 0.01; η*_p_*^2^ = 0.126). Moreover, extraversion was found to significantly moderate the relationship between the BDNF genotype and episodic memory performance (*p* = 0.03). Subsequent correlational analyses yielded a strong and significant correlation (*r* = 0.542; *p* < 0.005) between introversion and delayed episodic memory specific to BDNF_Met_ individuals. The present study suggests that introversion and the BDNF_Met_ variant synergistically interact to reduce episodic memory performance in healthy, young adults. These findings reaffirm that a more accurate explanation of cognitive variance can be achieved by looking at the synergistic effects of genotype and phenotype factors.

## 1. Introduction

The brain-derived neurotrophic factor (BDNF) belongs to the neurotrophin family and regulates cell survival, proliferation, and synaptic growth in many regions of the peripheral and central nervous system [1,2,3,4]. BDNF is widely expressed in the hippocampus [5] and plays an important role in human learning and memory processes through its modulating effect on synaptic changes, such as long-term potentiation (LTP) of hippocampal neurons [1,2,6].

A single functional nucleotide polymorphism (SNP) in the BDNF gene, resulting in a valine (Val) to methionine (Met) substitution at codon 66 in the prodomain (BDNF_Met_ or BDNF Val66Met), is significantly associated with reduced BDNF secretion in an activity-dependant manner along with impairment of intracellular trafficking [7]. Early studies on the cognitive consequences of this valine-to-methionine substitution have associated the Met allele with reduced indices of cognitive function, including general intelligence [8], working memory [9,10], speed of information processing [11], and episodic memory [12,13,14]. In support of this notion, functional neuroimaging studies highlighted that relative to BDNF_Val_ homozygotes, BDNF_Met_ carriers show a relative decrease in hippocampal activation during encoding and retrieval of declarative memory [13,14], which in turn alters declarative memory performance [12,15,16]. Moreover, reduced hippocampal volume is also a known consequence of carrying the BDNF_Met_ allele and the latter was associated with altered hippocampal function including declarative memory [17,18,19]. However, more recent reports on the effects of the BDNF_Met_ variant on cognitive function have yielded mixed results and the regulating role of this BDNF polymorphism on episodic memory has been challenged in recent years [20]. Episodic memory is a subtype of declarative memory that includes the personally-experienced and conscious recollection of life events [21]. The growing interest in studying the fate of BDNF_Met_ polymorphism partly stems from its high frequency in the Caucasian population (about 20%–30% carry at least one Met allele) [16,22], making any functional consequence of sizeable societal significance [23].

Another major factor in studying individual differences in cognitive performance, other than genetic vulnerability, is personality types. These are likely to be manifested in relatively stable and consistent patterns of cognition, emotion, motivation, and behavior, in response to a variety of eliciting stimuli [24]. There is now substantial consensus on the structure of personality in adulthood, with the most influential model being the Five-Factor Model of Personality [25]. According to this hierarchical model of trait structure, specific traits are organized in terms of five broad factors (neuroticism, extraversion, openness to experience, agreeableness and conscientiousness) that appear valid cross-culturally [26,27] and relatively stable across the lifespan [27]. Interestingly, among personality traits that influence cognitive performance, extraversion was associated with intelligence, especially with verbal ability [28,29,30]. Furthermore, a 25-year follow-up study in 4039 aging adults (aged 65 and older) showed that moderate extraversion was linked to lower risk of cognitive impairment in both case-control and co-twin designs [31]. This positive association between intelligence and extraversion is at least partially explained by the higher processing speed and assertiveness in extraverted individuals, which is advantageous for performance in most type of psychometric tests [29,32]. Conversely, introversion (i.e., low extraversion) seem to be a critical trait to predict reduced cognitive performance, mainly because of its likelihood to elicit test anxiety and lack of confidence [32,33].

In older adults (above the age of 60), extraversion has been positively associated with episodic memory, such that extraverted older adults tend to exhibit better free recall abilities relative to age-equivalent introverted individuals [29,34,35]. However, much less research has attempted to study the relation between extraversion and episodic memory performance in young adults. The major interest in studying factors that may interfere with episodic memory function at an older age stems from the clinical demonstration that episodic memory decline represents the commonest early symptom of the installation of Alzheimer’s disease [36,37,38,39]. However, one might wonder whether extraversion effects on episodic memory is age-dependent as opposed to being observed consistently throughout the lifespan.

In parallel, a genome-wide association (GWA) study on a genetically isolated population of 3972 individuals in Sardinia showed that the extraversion/introversion trait was selectively associated with the BDNF gene [40]. Interestingly, BDNF_Met_ carriers were found to be more introverted (and thus had a significantly lower score at the NEO-PI-R Extraversion scale) compared with BDNF_Val_ carriers [40]. These results were replicated in two independent, large cohorts from Italy (*n* = 1560) and the United States (*n* = 1131) [41].

While most studies have been interested in the distinct, predisposing roles of the common BDNF gene polymorphism and extraversion personality traits on episodic memory decline, very few studies have looked at the synergistic effects of genetic and personality factors to account for the significant variability in cognitive function on episodic memory performance. However, in a recent study from our group, middle-aged healthy adults who carried the BDNF_Met_ allele were found to be significantly more introverted, and among them, BDNF_Met_ carriers with the lowest extraversion scores were also those who obtained the lowest performance score on an episodic memory task [42]. These findings suggest that at least for middle-aged healthy adults, a more accurate explanation of cognitive variance can be achieved by looking at the synergistic effects genotype and phenotype factors.

In keeping with these findings in middle-aged healthy adults, the current study aimed to examine the potential relationship between the BDNF Val66Met polymorphism, the extraversion personality trait and episodic memory performance in a population of young healthy adults. We hypothesized that BDNF_Met_ carriers would be more introverted when compared to homozygous BDNF_Val_ carriers. Although debated in the literature, we hypothesized that BDNF_Met_ carriers would exhibit poorer episodic memory performance than BDNF Val homozygotes. Most importantly, it was hypothesized that episodic memory performance would be most impaired in BDNF_Met_ carriers who exhibit a more introverted personality type.

## 2. Results

### 2.1. Univariate Analyses of Variance

Univariate analyses of variance (ANOVA) were computed to assess the effect of BDNF polymorphism on extraversion as well as on neuropsychological tests (immediate and delayed word recalls). The ANOVA revealed that BDNF_Met_ carriers were significantly less extraverted (and therefore more introverted) than BDNF_Val_ carriers (*F*_1,73_ = 9.54; *p* < 0.01; η*_p_*^2^ = 0.126). The BDNF_Met_ variant, however, was not found to significantly change performance scores on any of the neuropsychological test including both immediate and delayed free recall of the RAVLT (immediate recall: *F*_1,73_ = 0.35; *p* = 0.61; η*_p_*^2^ = 0.006; delayed recall: *F*_1,73_ = 1.18; *p* = 0.29; η*_p_*^2^ = 0.021). Results from ANOVAs conducted for each neuropsychological test and personality traits are presented in Table 1.

### 2.2. Regression Analyses

**Figure 1:**
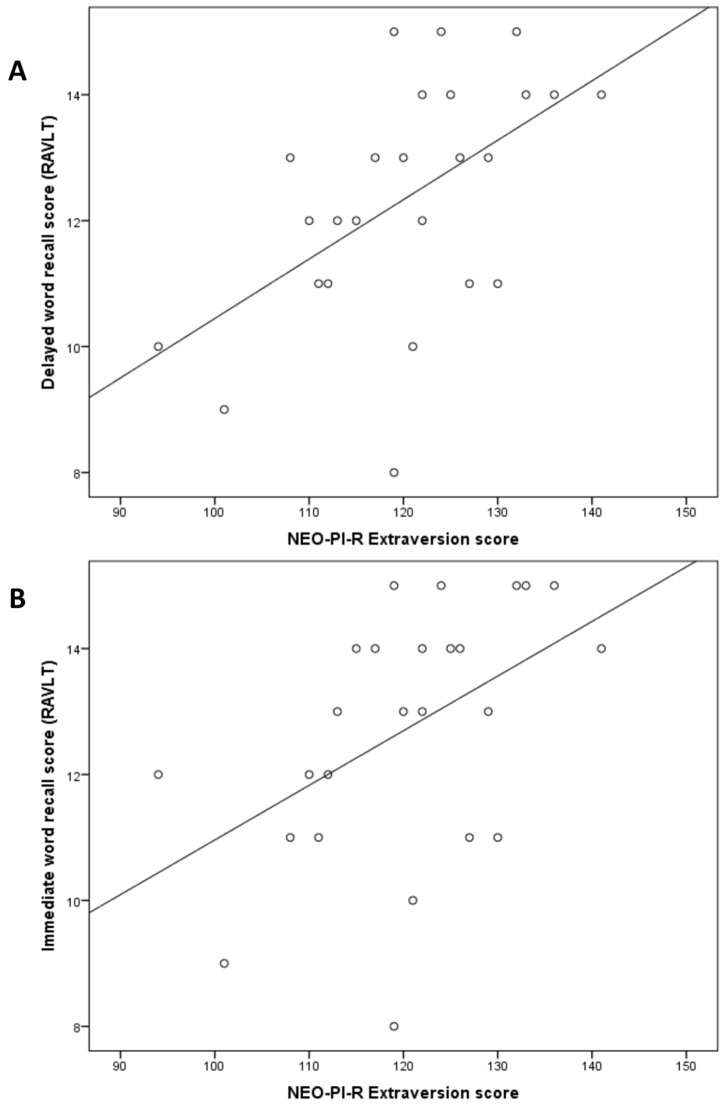
(**A**) Scatter plot illustrating the relation between the extraversion score at the NEO PI-R and delayed word recall performance in young healthy adults carrying at least one copy of the BDNF_Met_ allele; (**B**) Scatter plot illustrating the relation between the extraversion score at the NEO PI-R and immediate word recall performance in young healthy adults carrying at least one copy of the BDNF_Met_ allele.

A linear regression showed that extraversion significantly moderated episodic memory performance across BDNF genotypes (β = 0.312; *p* = 0.03). Further correlational analyses stratified by BDNF genotype showed that for homozygous BDNF_Val_ carriers, no significant correlation was found between scores on the NEO-PI-R Extraversion subscale and both immediate word recall score (*r* = −0.029; *p* = 0.87) and delayed word recall score (*r* = 0.096; *p* = 0.60). However, for the BDNF_Met_ carriers, extraversion was positively correlated with both immediate word recall (*r* = 0.479; *p* = 0.015) and delayed word recall (*r* = 0.542; *p* = 0.005) (Table 2; Figure 1). These results indicate that the modulating effects of extraversion on episodic memory performance are selective to BDNF_Met_ carriers. Other correlations stratified by BDNF genotype were also conducted to assess potential associations between extraversion and neuropsychological tests. Extraversion did not correlate with any of the other neuropsychological measures (Refer to Table 2).

## 3. Discussion

The present study found an association between polymorphism in the BDNF gene, extraversion and episodic memory performance in a population of young adults. In keeping with previous findings [40,41,42], BDNF_Met_ carriers scored significantly lower in extraversion (i.e., higher levels of introversion) when compared to BDNF_Val_ carriers. While no group difference by BDNF polymorphism was found for episodic memory performance, a significant and positive correlation was observed between extraversion and episodic memory performance for the young, BDNF_Met_ carriers, which was absent in age-equivalent BDNF_Val_ carriers. Further moderation analyses revealed that extraversion significantly moderated the relationship between BDNF polymorphism and episodic memory performance, in such a way that high levels of introversion significantly contribute the reduction in declarative memory performance in young, BDNF_Met_ carriers, but not in age-equivalent BDNF_Val_ carriers.

The main finding of this study is that the extraversion personality trait interacts with the BDNF_Met_ polymorphism to alter episodic memory performance among young adults. Consistent with results obtained in a sample of 135 healthy young adults [43], the present study found an absence of BDNF_Met_ variant effect alone on episodic memory performance. Age-associated synaptic plasticity efficiency loss may provide an explanation as to why BDNF genotype only affects episodic memory performance later in life [44]. Accordingly, more efficient synaptic plasticity in early adulthood could compensate for hippocampal vulnerabilities associated with the BDNF_Met_ polymorphism. In addition, knowing that episodic memory performance and BDNF levels are both known to decrease steadily with the normal ageing process [45], young adults carrying the Met allele could find themselves at a lower risk of episodic memory alterations due to the abundance of BDNF [46]. Another possible explanation for this age-dependent effect of the BDNF_Met_ variant on episodic memory performance could be that extraversion, a moderating factor of the relationship between genotype and memory performance in the present study, is considerably shaped by experiences over the lifespan, especially by social and stressful experiences, and is negatively associated with age [47,48], in part because of the lower social stimulation found in older adults [35]. In light of these results, it appears likely that among introverted BDNF_Met_ carriers, older adults exhibit, on average, a higher level of introversion than young adults, which could exacerbate between-group differences on memory performance in older adults. Thus, the present study suggests that individual differences in memory seen in middle-aged introverted BDNF_Met_ carriers [42] may be present from their early age. A longitudinal follow-up study would be helpful to assess whether age-associated memory performance reductions could be linked to concomitant changes in personality across the lifespan.

The combined genetic-personality trait effect on memory performance can potentially be explained by the synergistic deleterious effects of introversion and the BDNF_Met_ variant on poor adaptation to environmental stress [49,50]. Indeed, it is well known that high stress levels indirectly lead to poorer memory processes via stress dysregulation effects on hippocampal long-term potentiation [51,52,53]. In BDNF_Met_ knock-in mice, higher stress is found to deplete BDNF availability, which alters memory processes [54,55]. In humans, it was shown that Met allele carriers tend to exhibit higher anxiety levels, which indirectly affect memory processes through HPA stress response dysregulation [49,56,57]. Furthermore, Met allele carriers tend to benefit from less social support and they tend to be less engaged than BDNF Val homozygotes in social interactions [58], which could lead to higher stress levels and thus, to poorer memory performance. In agreement with these findings, higher levels of introversion found in Met allele carriers from this study were shown to significantly associate with lower performance scores in an episodic memory task. The current study results are in line with a previous report suggesting that extraversion is protective against stress outcomes [59] and is associated with adjustment to stress, life satisfaction and resilience [50,60]. Indeed, extraverts seek more social support/interaction than introverts [61,62,63] and engage their social networks under stressful conditions, which help them to efficiently cope with stress [63,64,65]. It is therefore possible that the quality and abundance of social interactions among extraverts provided them with plentiful opportunities to find themselves in a learning context where memory skills can be further developed.

In addition, studies show that extraverts experience more positive affects [50,66,67,68] while introverts experience more negative emotions [69]. Considering that higher positive affects experienced during encoding processes generate emotional contextual markers that are associated with event memory traces to be remembered, it is possible that a higher level of extraversion contributes to better memory performance [21,34,70]. The BDNF Met allele and introversion could be conceptualized as individual vulnerabilities that possibly predispose the individual to lower social support/interactions, poorer adaptation to stress and less positive affect, which may in turn alter memory processes. Thus, the present results extend previous findings as it shows that low extraversion interact with the BDNF Met variant to reduce episodic memory performance throughout the lifespan.

From a clinical perspective, as extraversion can be conceptualized as a buffer in the trajectory of age-related episodic memory decline [35,71] and other cognitive functions, the addition of a personality assessment in standard neuropsychological practice could further refine our clinical understanding of longitudinal changes of cognitive function. Moreover, the BDNF_Met_ x introversion association with poorer episodic memory performance found in the current study also highlights the need for future studies to carefully take into account the modulating role of environmental factors such as stress and social interactions on memory processes.

Although this study is the first to investigate the effect of personality and BDNF on episodic memory performance in a population of young adults, it is not without limitations. A large-scale study will be helpful to replicate the current study findings due to its restricted sample size. Serum levels of BDNF could also reveal to be informative if we are to further investigate the association between memory performance and available BDNF levels.

## 4. Materials and Methods

### 4.1. Participants

All 75 participants in this study were collegial or university-level Caucasian students from French-Canadian origins, with an average education level of 14.38 years (SD = 1.61). The sample was composed of 38 women (51.0%) and 37 men (49.0%) aged between 18 and 26 years (mean age = 20.71; SD = 1.81) recruited consecutively through newspaper advertisements and posters located throughout our University institution. Participants who took part in this study were those who were not excluded after having been screened for the following exclusion criteria, which are known to influence cognitive performance: A history of alcohol and/or substance abuse, psychiatric illness (e.g., depression, anxiety), developmental disorders (e.g., ADHD, autism spectrum disorders), learning disabilities (e.g., dyslexia), neurological history (e.g., seizure, epilepsy, central nervous system neoplasm, brain tumour), a history of traumatic brain injury, and having undergone cognitive testing during the year.

In this sample, there were 4 (5.3%) homozygous Met allele carriers, 22 (29.3%) heterozygous Met allele carriers and 49 (65.3%) homozygous Val allele carriers. Due to the rare occurrence of the Met allele in a homozygous state, carriers of this genotype were added to the group of heterozygous Met allele carriers. Participants were subdivided into two groups. The first group consisted of homozygous Val allele carriers (referred to as BDNF_Val_; *n* = 49) and the second group consisted of both heterozygous and homozygous carriers of the Met allele (referred to as BDNF_Met_; *n* = 26). Groups were equivalent according to age, level of education and sex (refer to Table 1). One participant had to be excluded from further analyses after rejection for outliers (Grubb’s test) at the NEO-PI-R extraversion subscale.

### 4.2. Procedure

Selected participants were tested at the Université du Québec à Trois-Rivières. All participants provided written informed consent before undergoing the study procedure. Saliva samples were taken from which the DNA could be extracted and genotyped. Participants were administered standard, neuropsychological tests and questionnaires and the study was approved by the local ethics committee of the Université du Québec à Trois-Rivières (Approved date: 1 May 2014; project ID code: CER-14-176-07.11).

#### 4.2.1. Neuropsychological Testing

The neuropsychological test battery included the Color-Word Interference test, the Trail Making Test and the Verbal Fluency Test of the D-KEFS, assessing general frontal lobe function/executive functions [72]. The Rey Complex Figure Test (RCFT), assessing visual-constructional ability, visual-spatial organization and visual memory [73] was also administered. Both immediate and delayed recall conditions of the Rey Auditory Verbal Learning Test (RAVLT) were administered to evaluate verbal episodic memory. Briefly, the RAVLT is divided into two lists (A and B), each made up of 15 unrelated nouns. List A is first read out loud five times to the participant, each time followed by free recall. Then, List B (interference) is read, followed by free recall (B). Participants are then asked to recall List A immediately (immediate recall) and 20 minutes later (delayed recall) [74]. The RAVLT has been shown to be reliable, with test-retest reliabilities between 0.63 and 0.84 for the immediate recall trials and between 0.57 and 0.78 for the delayed recall trials [75].

#### 4.2.2. Questionnaires

First, a self-reported questionnaire (General health questionnaire) was administered to screen for medical exclusion criteria known to influence brain function. In addition, the Beck Anxiety Inventory questionnaire and the Beck Depression Inventory were administered to exclude participants who scored above the pre-established clinical threshold for anxiety and depression. Furthermore, the Revised NEO Personality Inventory (NEO-PI-R) [76] was used to assess the five personality domains (the Big Five): neuroticism, extraversion, openness to experience, agreeableness and conscientiousness. The NEO PI-R has a robust factor structure replicated in more than 50 cultures [77]. Moreover, all five subscales provide a reliable measure of its corresponding construct, as evidenced by high reliability coefficients (from 0.75 to 0.89) and test-retest reliabilities coefficients (from 0.63 to 0.91) [76].

#### 4.2.3. Genotyping

We performed DNA extraction from the buffy coat using Qiagen EZ1 DNA kit (Hilden, Germany). A PCR method was followed by pyrosequencing to obtain genotype profiling of BDNF rs6265 (Val66Met) polymorphism. The following primer pairs were used for PCR-based amplification: forward biotin 5′-GGACTCTGGAGAGCGTGAAT-3′ and reverse 5′-CCGAACTTTCTGGTCCTCATC-3′. Genomic DNA (250–500 ng) was amplified with 10 pM/of each primer, 1× PCR buffer (Quiagen kit), 0.4 mM dNTP, 1.0 mM MgCl_2_, and 0.01 U of Quiagen Taq polymerase. We then used a Biometra Tprofessional Basic thermocycler (Biometra, Göttingen, Germany) to conduct a 35-cycle amplification stage preceded by a 2-min hot start at 95 °C and followed by a final 4-min extension to the last cycle at 72 °C. Each cycle consisted of the following steps: 30 s at 95 °C, 30 s at 61.2 °C and 1 min at 72 °C. A 1.2% agarose gel was used to visualize PCR products. The Val66Met polymorphism was sequenced with a pyrosequencing protocol [78] routinely used in our laboratory with a slight modification using the sequencing oligomer 5′-GCTGACATTTCGAACA-3′. The sequence to analyze was: CA/GTGATAGAAGAG.

### 4.3. Statistical Analyses

All values are expressed as means (SDs). Data were analyzed with SPSS 21 (SPSS, Chicago, IL, USA). The significance level was set at α = 0.05, bilaterally. Groups were subjected to standard descriptive statistics. Effect sizes for mean differences were estimated with partial eta squared (η*_p_*^2^). Between-group differences in neurocognitive test results and NEO-PI-R subscales scores were assessed using univariate analyses of variance (ANOVA). For each group separately, two-tailed Pearson correlations were computed between extraversion score and neuropsychological test scores. A linear regression analysis was performed to test the moderating effect of introversion on episodic memory performance in BDNF_Met_ carriers.

## 5. Conclusions

Despite its limitations, this study is the first to investigate the effect of personality and BDNF genotype on episodic memory performance in a population of young adults. Our findings show that introversion and BDNF_Met_ polymorphism interact to significantly affect episodic memory performance in young adults. Results from this study also suggest that reduced episodic memory found in middle-aged introverted BDNF_Met_ carriers are present throughout the lifespan. Furthermore, we observed that in young adults, BDNF_Met_ polymorphism alone was not associated with episodic memory performance, therefore reaffirming the pertinence to consider personality, especially the extraversion trait, along with genetic factors to account for cognitive variance at all ages.

## Figures and Tables

**Table 1:** Participant’s demographics and genotype effects on cognition and personality.

Variables of Interest	BDNF Genotype		*F*	*p*
	BDNF_Met_ (*n* = 25)	BDNF_Val_ (*n* = 49)		
Age (year)	20.96 (1.94)	20.58 (1.66)	0.79	0.38
Education (year)	14.81 (1.72)	14.16 (1.53)	2.818	0.09
BAI	5.03 (4.85)	4.79 (4.25)	0.05	0.83
BDI	3.07 (2.76)	3.73 (2.97)	0.88	0.35
Personality NEO-PI-R				
Neuroticism	81.76 (17.14)	80.21 (21.18)	0.103	0.75
Extraversion	120.28 (10.87)	131.05 (12.44)	9.54	0.01 *
Openness	101.04 (13.79)	105.79 (16.31)	1.60	0.21
Agreeableness	120.63 (18.21)	121.78 (12.49)	0.10	0.75
Conscientiousness	119.11 (24.19)	118.52 (16.37)	0.02	0.90
RAVLT				
Word Immediate recall	12.19 (2.77)	11.81 (2.02)	0.46	0.50
Word Delayed recall	12.06 (2.30)	11.38 (2.56)	1.28	0.26
RCFT				
Immediate recall	23.06 (6.16)	24.03 (5.21)	0.51	0.47
Delayed recall	22.92 (6.05)	24.07 (5.27)	0.73	0.40
D-KEFS Verbal Fluency Test				
Condition 3 Total switching accuracy	13.47 (2.19)	12.71 (2.69)	1.53	0.22
Set-Loss Errors	0.74 (0.85)	0.89 (1.07)	0.38	0.54
D-KEFS Trail Making Test				
Condition 4 Total Time	50.62 (12.01)	50.78 (14.23)	0.002	0.96
Condition 4 Total Errors	0.61 (0.75)	0.64 (0.81)	0.024	0.87
D-KEFS Color-Word Interference Test				
Condition 4 Total Time	49.71 (8.60)	49.97 (12.45)	0.009	0.93
Condition 4 Total Errors	1.06 (0.72)	1.31 (1.29)	0.84	0.36

BDNF: brain-derived neurotrophic factor; Met: methionine; Val: valine; RAVLT: Rey Auditory Verbal Learning Test; RCFT: Rey Complex Figure Test; SDMT: Symbol Digit Modalities Test; D-KEFS: Delis-Kaplan Executive Function System. Numbers in parentheses represent standard deviations values. * *p* < 0.05.

**Table 2:** Correlation between extraversion and cognition by genotype.

**BDNF_Met_**	***R***	***p***	***n***
RAVLT			
Word Immediate recall	0.479	0.015 *	25
Word Delayed recall	0.542	0.005 *	25
RCFT			
Immediate recall	0.151	0.46	25
Delayed recall	0.182	0.37	25
D-KEFS Verbal Fluency Test			
Condition 3 Total switching accuracy	0.256	0.21	25
Set-Loss Errors	0.249	0.22	25
D-KEFS Trail Making Test			
Condition 4 Total Time	0.321	0.11	25
Condition 4 Total Errors	−0.014	0.94	25
D-KEFS Color-Word Interference Test			
Condition 4 Total Time	−0.131	0.52	25
Condition 4 Total Errors	−0.024	0.91	25
**BDNF_Val_**	***R***	***p***	***n***
RAVLT			
Word Immediate recall	−0.035	0.81	49
Word Delayed recall	0.125	0.39	49
RCFT			
Immediate recall	0.068	0.64	49
Delayed recall	0.050	0.73	49
D-KEFS Verbal Fluency Test			
Condition 3 Total switching accuracy	0.172	0.24	49
Set-Loss Errors	0.085	0.56	49
D-KEFS Trail Making Test			
Condition 4 Total Time	−0.201	0.17	49
Condition 4 Total Errors	−0.053	0.72	49
D-KEFS Color-Word Interference Test			
Condition 4 Total Time	0.016	0.91	49
Condition 4 Total Errors	−0.216	0.14	49

BDNF: brain-derived neurotrophic factor; Met: methionine; Val: valine; RAVLT: Rey Auditory Verbal Learning Test; RCFT: Rey Complex Figure Test; SDMT: Symbol Digit Modalities Test; D-KEFS: Delis-Kaplan Executive Function System. Numbers in parentheses represent standard deviations values. * *p* < 0.05.
